# On non-adjacent letter repetition and orthographic processing: Lexical decisions to nonwords created by repeating or inserting letters in words

**DOI:** 10.3758/s13423-020-01837-1

**Published:** 2020-11-24

**Authors:** Emilia Kerr, Jonathan Mirault, Jonathan Grainger

**Affiliations:** Laboratoire de Psychologie Cognitive, CNRS, Aix-Marseille University, 3 place Victor Hugo, 13331 Marseille, France

**Keywords:** Letter repetition effects, Orthographic processing, Word recognition, Reading

## Abstract

Informal observation suggests that it is harder to notice the spelling mistake in “silencne” than “silencre.” This concurs with current evidence that non-adjacent letter repetition in correctly spelled words makes these words harder to recognize. One possible explanation is provided by open-bigram coding. Words containing repeated letters are harder to recognize because they are represented by fewer bigrams than words without repeated letters. Building on this particular explanation for letter-repetition effects in words, we predicted that nonwords in a lexical decision task should also be sensitive to letter repetitions. In particular, we examined two types of nonwords generated from the same baseword: (1) nonwords created by repeating one of the letters in the baseword (e.g., silence => silencne); and (2) nonwords created by inserting a letter that is not present in the baseword (e.g., silencre). According to open-bigram coding, nonwords created by repeating a letter are more similar to their baseword than nonwords created by inserting a letter, and this should make it harder to reject letter repetition nonwords than letter insertion nonwords. We put these predictions to test in one on-line pilot study (n=31), one laboratory experiment (n=36), and one follow-up on-line experiment (n=40) where we manipulated the distance between repetitions (one, two, three, or four letters). Participants found it harder to reject repetition nonwords than insertion nonwords, and this effect diminished with increasing distance.

## Introduction

Some readers might have already noticed that detecting the spelling mistake indicated by a red underline in MS Word is particularly difficult when that mistake is caused by the repetition of a letter that is already in the word (e.g., silencne, repetititon). Among different possible accounts of this phenomenon, there is one model of orthographic processing, the open-bigram model (Grainger & van Heuven, 2004; Whitney^1^, ^2001^), which predicts this perceived difficulty. The core mechanism of this model is the way that location-invariant letter order is encoded – via an unordered set of ordered contiguous and non-contiguous letter pairs referred to as “open-bigrams” (e.g., word = od, wd, or, wo, wr, rd). According to this coding scheme, there is only one open-bigram in the nonword “silencne” that is incompatible with the incorrectly written word “silence” – that is the bigram “cn.” On the other hand, if the typographical error is caused by the insertion of a letter that is not already present in the targeted word (e.g., silencre), then the number of incompatible open-bigrams is greater (er, nr, cr, re), hence making it easier to detect the error (we apply the parameters of the Grainger & van Heuven, 2004, model in these calculations, see also Appendix A). In the present study we examine whether this anecdotal evidence finds support in a more a more tightly controlled empirical investigation. First, we summarize the current evidence for an impact of letter repetition on reading behavior, before describing the present manipulation and the predictions of alternative models of letter-position coding concerning this manipulation.

Although letter-repetition effects have been investigated using various paradigms in the past (e.g., Bjork & Murray, 1977; Gomez, Ratcliff, & Perea, 2008; Kanwisher, 1991; Mozer, 1989), Schoonbaert and Grainger (2004) were among the first to investigate effects of within-word letter repetition in a reading paradigm (see Harris & Morris, 2000, for a demonstration of between-word repetition effects referred to as “orthographic repetition blindness”). This was an important step forward in an attempt to reveal an impact of letter repetition on the processes involved in visual word recognition. Schoonbaert and Grainger’s (2004) study produced mixed findings. On the one hand they did report that target words with repeated letters were harder to respond to in a lexical decision task than words with no letter repetitions. On the other hand, they found that masked primes formed by removing a repeated letter in a target word (e.g., balnce –BALANCE) were no more effective than primes formed by removing a non-repeated letter (e.g., balace – BALANCE). Furthermore, in an unprimed lexical decision task, nonword targets formed by removing a repeated letter from a real word (e.g., BALNCE) were not any harder to respond to than nonwords formed by removing a non-repeated letter (e.g., BALACE). It is this ensemble of letter-repetition effects for word targets and null effects for nonword primes that motivated the parameters implemented in the Grainger and van Heuven (2004) model. By simply imposing a limit on the number of letters that can intervene between the constituent letters of an open-bigram, set to two in the Grainger and van Heuven model, then the model could account for the complete set of findings.

One primary inspiration for the present study is the more recent work of Trifonova and Adelman (2019), which importantly renewed interest in letter-repetition effects, and crucially brought attention to the difficulty that a number of prominent models of orthographic processing have in accounting for such effects. Trifonova and Adelman (2019) performed regression analyses on several mega-studies of lexical decision and word naming (Balota et al., 2007; Brysbaert et al., 2016; Ferrand et al., 2010; Keuleers et al., 2012). They found a small but significant inhibitory influence of repeated letters when the repetition did not involve adjacent letters. The effects were modulated by the distance separating the repeated letters, being strongest with one to three intervening letters. Here we simply aimed to provide a further test of one possible explanation of these letter-repetition effects, that is intrinsically tied to the core principles of open-bigram coding. After excluding the special case of adjacent letter repetitions, often referred to as “double letters” (e.g., Caramazza & Miceli, 1990; Fischer-Baum, 2017), open-bigram coding offers a principled^2^ account of non-adjacent letter-repetition effects, since letter repetition affects the number of open-bigrams that are generated by a written word, and open-bigram activation is the main mechanism governing activity in whole-word orthographic representations during silent reading (Grainger & Ziegler, 2011; Snell, van Leipsig, Grainger, & Meeter, 2018).^3^ Crucially, for the present work, in the Grainger and van Heuven (2004) model the number of incompatible open-bigram representations plays a role via inhibitory connections between bigrams and whole words.

In the present study we apply what might arguably be the simplest of methodologies to reveal the effects of non-adjacent letter repetition on orthographic processing. We adopt a methodology that has already been successfully applied to investigate transposed-letter effects (Andrews, 1996; Bruner & O’Dowd, 1958; Chambers, 1979; Frankish & Turner, 2007; O’Connor & Forster, 1981; Perea, Rosa, & Gomez, 2005). The basic finding here is that nonwords created by transposing two letters in a real word (e.g., gadren – derived from “garden”) are harder to classify as nonwords in a lexical decision task compared with nonwords formed by substituting two letters in a real word with different letters (e.g., gatsen). Building on the anecdotal evidence that it might be harder to detect typographical errors when these involve the erroneous repetition of a letter that is already part of the word, here we used performance to different types of nonword targets in a lexical decision task as a means to investigate this phenomenon in a controlled laboratory setting. We compared performance to two types of nonwords generated from the same baseword: (1) nonwords created by repeating one of the letters in the baseword (e.g., silencne); and (2) nonwords created by inserting a letter that is not present in the baseword (e.g., silencre). According to open-bigram theory, the “repeated letter” nonwords are more similar to their basewords than the “inserted letter” nonwords, and therefore should be harder to reject as a nonword in the lexical decision task. Thus, for example, the repeated letter nonword “silencne” only contains a single open-bigram (cn) that does not occur in the baseword “silence.” On the other hand, the nonword “silencre,” formed by inserting a letter that is not in the baseword, contains multiple open-bigrams that do not occur in the baseword (see Davis, Perea, & Acha, 2009, for prior evidence in favor of such inhibitory effects on the processing of nonwords in lexical decision). Therefore, it should be harder to reject “silencne” as a nonword compared with “silencre” because it is more similar to its baseword. We put these predictions^4^ to test in one laboratory experiment (Experiment 1) and one on-line experiment (Experiment 2) that provides a controlled investigation of the effects of distance between letter repetitions.

## Experiment 1

### Methods

#### Participants

Thirty-six native speakers of French (29 female, mean age 21.8 years (SD = 3.4)) were recruited to complete the experiment in the laboratory setting. All reported having no neurological, language, or reading disorders. Participants were naïve to the purpose of the experiment. Ethics approval was obtained from the Comité de Protection des Personnes SUD-EST IV (No. 17/051). A power analysis with the R package SIMR (Green & MacLeod, 2016) performed on the data of an on-line pilot study testing the same stimuli as Experiment 1 (20 simulations) confirmed that the number of participants we recruited provided ample power for both response time (> 80% power reached with n=19) and error rate models (>80% power reached with n=9).

#### Design and stimuli

100 French words (see Appendix B) were selected from the Lexique database (version 3.83; New, Pallier, Brysbaert, & Ferrand, 2004) that were between 8 and 12 letters in length (mean 8.68, SD 0.87) and did not contain accented letters. They were all relatively high-frequency singular nouns (mean = 4.20 Zipf, SD = 0.47: van Heuven, Mandera, Keuleers, & Brysbaert, 2014), and had an average OLD20^5^ value of 2.64 (SD = 0.40: Yarkoni, Balota, & Yap, 2008). From these 100 basewords, two types of pronounceable and orthographically legal nonwords were created: (1) nonwords that contained a repeated letter from the baseword, e.g., *adjudant – adjudtant*; (2) nonwords that contained a foreign letter inserted in the baseword, e.g., *adjudant – adjudlant*. For both types of nonwords, the repeated or inserted letter occupied the same position in the baseword, varied across all internal positions across the different basewords. The repeated/inserted letter was two to four positions away from the letter that was repeated (i.e., separated by one, two, or three intervening letters) and was never located in the initial or final position. Average letter frequency, calculated using lowercase token frequencies in French (New & Grainger, 2011), was 9.49 Zipf (SD = 0.27) for the repeated letters and 9.38 Zipf (SD = 0.30) for the inserted letters. Letter frequency was included as a covariate in the statistical analyses. Since some basewords already contained repeated letters (47 out of 100, and mostly vowels), these letters were never involved in the repetition/insertion manipulation that always involved a consonant. Whether or not a baseword contained repeated letters was included as a covariate in the statistical analyses. The main factor was “type of nonword” – repetition versus insertion, and this was counterbalanced across participants using a Latin-square design. That is, two lists were created so that every participant was presented with a nonword derived from a given baseword in only one of the two conditions. OLD20 was used to control for differences in orthographic similarity to real words across the two sets of nonwords (mean = 3.25, SD = 0.41 in the repetition condition; mean = 3.27, SD = 0.42 in the insertion condition). These values were added as a covariate in the statistical analyses. Additionally, another 100 French words were selected from the Lexique database (with the same constraints as the basewords) for the purposes of the lexical decision task. The responses to these words were not analyzed.

#### Procedure

Participants were engaged in a lexical decision task where they were asked to indicate as accurately and as rapidly as possible whether the stimulus presented on the screen was a real word or not. Prior to the experiment, all participants signed a written consent form. Participants were comfortably seated in a testing room in front of a computer screen at a distance of approximately 70 cm. Stimuli were presented in lowercase letters on a computer monitor controlled by OpenSesame (version 3.1.9, Mathôt, Schreij, & Theeuwes, 2012). Each trial began with a fixation cross presented in the center of the screen for a random duration from 250 to 350 ms followed by the target word (a nonword or a real word) that stayed on the screen until the response was made. After each trial a feedback dot was provided in green (correct) or red (incorrect) presented for 300 ms. The intertrial interval was 200 ms. Prior to the main experiment, ten practice trials were completed by participants in order to familiarize them with the procedure. In the main experiment each participant completed 200 trials – 100 nonwords and 100 real words. Trial presentation was randomized with a different random order per participant. The experiment lasted approximately 10 min.

### Results

We computed response accuracy and response time (RT: the duration between the onset of the presentation of the target and the keyboard response) for correct responses. Participants performed with an average accuracy of 86.1% (SD = 34.5). We used Linear Mixed Effects Model (LME) to analyze RTs and Generalized (logistic) Linear Mixed Effects Model (GLME) to analyze response accuracy, with participants and items as crossed random effects (Baayen, Davidson, & Bates, 2008; Barr, Levy, Scheepers, & Tily, 2013). The models were fitted with lmer (for LME) and the glmer (for GLME) functions from the lme4 package (Bates, Maechler, Bolker, & Walker, 2015) in the R statistical computing environment (version 3.3.1, R Core Team, 2017). We report regression coefficients (*b*), standard errors (SE), and *t*-values (for LMEs) or *z*-values (for GLMEs). Fixed effects were deemed reliable if |t| or |z| > 1.96 (Baayen, 2008). We used the control condition as the reference. RTs were inverse-transformed (-1,000/RT) prior to analysis. We used the maximal random structure model that converged (Barr et al., 2013), and this included by-participant and by-item random intercepts in all analyses that we report.

#### Response times

Prior to the analysis, we excluded incorrect responses (10.1%), leaving a dataset of 2,977 observations. RTs beyond 2.5 SD from the grand mean were removed before analysis (2.78%), leaving a total of 2,894 data points. RTs were significantly slower (*b* = 0.06, SE = 0.029, *t* = 2.2) to nonwords with a repeated letter (M = 875 ms, 95% CI = 31) compared to nonwords with a foreign inserted letter (M = 841 ms, 95% CI = 30).

#### Error rates

Based on 3,312 observations we observed a significant effect of Type of Nonword (*b* = 1.2, SE = 0.31, *z* = 3.8), with error rates being significantly higher in the repetition condition (M =14.5%, 95% CI = 2.9) compared to the insertion condition (M = 5.7%, 95% CI = 3.2).

### Discussion

Experiment 1 provided clear support for our hypothesis that nonwords created by repeating a letter that is already present in a given baseword word (e.g., *silencne* from the baseword *silence*) are harder to reject compared with nonwords created by inserting a letter that is not present in the baseword (e.g., *silencre*). The repeated letters were separated by at least one letter (i.e., no adjacent repetitions) and fewer than four letters. In order to provide a preliminary analysis of the effects of distance, we performed post hoc analyses including distance as a factor. There were seven items for distance 1, 50 items for distance 2, and 43 items for distance 3. The repetition effect was 52 ms in RTs and 10.5% in errors for distance 1, 56 ms in RTs and 12.4% in errors for distance 2, and 6 ms and 4.1% for distance 3. The interaction between repetition and distance was significant for errors (*b* = -0.6, SE = 0.22, *z* = 2.78). On the basis of these preliminary findings we decided to run an experiment manipulating distance.

## Experiment 2

### Methods

#### Participants

Forty native speakers of French (15 males, mean age 27.5 years (SD = 11.6)) were recruited to complete the experiment online. All reported having no neurological, language, or reading disorders. Participants were naïve to the purpose of the experiment.

#### Design and stimuli

200 French words (see Appendix C) were selected from the Lexique database (version 3.83; New, Pallier, Brysbaert, & Ferrand, 2004) that were eight letters in length and did not contain accented letters. They were all relatively high-frequency words (mean 4.25 Zipf, SD = 0.41), and had an average OLD20 value of 2.28 (SD = 0.39). From these 200 basewords, two types of pronounceable and orthographically legal nonwords were created as in Experiment 1. The main factors were (1) Type of Nonword – repetition vs. insertion, and (2) Distance (i.e., the repeated/inserted letter was separated by one, two, three, or four intervening letters, 50 nonwords per distance and type of nonword). This was counterbalanced across participants using a Latin-square design. The average frequency of the repeated letters was 9.43 Zipf (SD = 0.34) and 9.43 Zipf (SD = 0.30) for the inserted letters. Additionally, another 200 French words of nine letters in length were selected from the Lexique database for the purposes of the lexical decision task. The responses to these words were not analyzed.

#### Procedure

Participants were engaged in a lexical decision task as in Experiment 1. The stimulus presentation was controlled using an in-house script.

### Results

The analysis methods performed in Experiment 2 were the same as in Experiment 1. Participants performed with an average accuracy of 92.5 % (SD = 26.3). Mean RTs and error rates with differences between the conditions (repetition effects) are presented in Table 1.

**Table 1:** Mean response times (RTs, ms) and percent errors (%ER) per condition in Experiment 2, and repetition effects (difference between the repetition and insertion conditions) per distance

	Distance 1		Distance 2		Distance 3		Distance 4	
	RT	%ER	RT	%ER	RT	%ER	RT	%ER
Repetition	845 (39)	10.0 (3.9)	839 (38)	13.2 (3.8)	825 (37)	12.5 (3.8)	817 (36)	8.8 (3.9)
Insertion	758 (35)	2.1 (4.0)	792 (36)	3.0 (4.0)	803 (36)	5.0 (4.0)	780 (34)	5.0 (4.0)
Difference	**87**	**7.9**	**47**	**10.2**	**22**	**7.5**	**37**	**3.8**

*Note.* 95% CIs are shown in parentheses

#### Response times

Prior to the analysis, we excluded incorrect responses (7.5%), leaving a dataset of 7,401 observations. RTs beyond and below 2.5 SD from the grand mean were removed before analysis (0.96%), leaving a total of 7,330 data points. RTs were found to be significantly slower (*b* = 0.12, SE = 0.02, *t* = 6.8) to nonwords with a repeated letter (M = 834 ms, 95% CI = 19.1) compared to nonwords with a foreign inserted letter (M = 785 ms, 95% CI = 17.9). Crucially, the Type of Nonword × Distance interaction was significant (*b* = 0.02, SE = 0.007, *t* = 3.12). Repetition effects were significant for distances 1, 2 and 4 (*b* = 0.01, SE = 0.02, *t* = 7.75; *b* = 0.07, SE = 0.01, *t* = 4.92; *b* = 0.07, SE = 0.01, *t* = 5.08), but not for distance 3 (*b* = 0.02, SE = 0.017, *t* = 1.36).

#### Error rates

Based on 8,000 observations, we observed a significant effect of the Repetition factor (*b* = 2.1, SE = 0.263, *z* = 8.01), with error rates being significantly higher in the repetition condition (M =11%, 95% CI = 2) compared to the insertion condition (M = 3.85%, 95% CI = 3). The Type of Nonword × Distance interaction was significant (*b* = 0.34, SE = 0.009, *z* = 3.9). Overall, the effects of repetition diminished with increasing distance, but were significant for all four distances (*b* = 1.9, SE = 0.3, *z* = 6.9; *b* = 1.6, SE = 0.20, *z* = 8.06; *b* = 1.1, SE = 0.18, *z* = 6.07; *b* = 0.6, SE = 0.18, *z* = 3.4).

#### Inverse efficiency

**Fig. 1:**
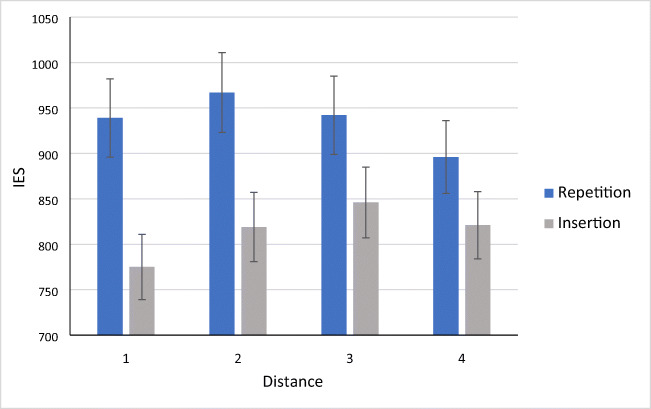
Average inverse efficiency scores (IES: response time/probability correct) per condition in Experiment 2. Note that different basewords were used to create the nonwords tested in the different distance conditions, so only the comparison between the repetition and insertion conditions per distance is relevant here. Differences in IES per distance are 164, 148, 96, 75. Error bars are 95% CIs

Given the theoretical importance of the distance factor (see Appendix A) and given the conflicting pattern in RTs and error rates (see Table 1, where opposite effects of repetition can be seen in RTs and errors across distances 1 and 2, and 3 and 4), we decided to compute inverse efficiency scores (IES) that combine RTs and error rates per condition and per participant. Inverse efficiency is obtained by dividing mean RT by probability correct (e.g., a mean RT of 500 ms with 90% accuracy gives 500 / 0.9 = 556). The condition means are shown in Fig. 1. A by-participant ANOVA was performed on these data with Type of Nonword (repetition vs. insertion) as a factor and Distance (1–4) as a covariate. The main effect of Type of Nonword was significant (F(1,158) = 74.53, *p* < .001), as was the interaction between Type of Nonword and Distance (F(1,158) = 15.18, *p* < .001). As can be seen in Fig. 1, the effects of Type of Nonword diminished with increasing Distance.

### Discussion

The results of Experiment 2 perfectly replicate the letter-repetition effect reported in Experiment 1 and enable a more precise evaluation of the impact of the distance (in number of letters) separating the repeated letters. This impact is best seen in Fig. 1, which reports a combined measure of RTs and error rates in the form of inverse efficiency. Here it is clear that the effects of non-adjacent letter repetition gradually diminish as the distance between the repeated letters increases. We examine the theoretical importance of these findings below.

## General discussion

In the present study we compared performance of two types of nonwords in a lexical decision task. These two types of nonwords were generated from the same set of basewords (e.g., silence) by either repeating a letter that is already present in the word (e.g., silencne) or by inserting a letter that is not present in the word (e.g., silencre), with the repeated letters separated by at least one letter and fewer than four letters in Experiment 1, and one to four letters in Experiment 2. In both experiments we observed that the repetition nonwords were harder to classify as such than the insertion nonwords, in terms of both RTs and error rates. This is a novel finding that we expect will provide important additional constraints on models of letter position coding and orthographic processing.

The letter-repetition effect on nonword processing was predicted by Grainger and van Heuven’s (2004) model of orthographic processing. The core mechanism of this model is the way in which location-invariant letter order information is encoded via a bag of contiguous and non-contiguous ordered pairs of letters (open-bigrams: Grainger & Whitney, 2004; Whitney, 2001). Thus, in the version described by Grainger and van Heuven (2004), a word such as “silence” is represented by the unordered set of the following open-bigrams: si, sl, se, il, ie, in, le, ln, lc, en, ec, ee, nc, ne, ce. This model provided a straightforward account of the findings of Schoonbaert and Grainger (2004) and Trifonova and Adelman (2019) that words with non-adjacent repeated letters are harder to respond to in the lexical decision task than words that do not contain letter repetitions. Words with letter repetitions activate fewer open-bigrams, hence reducing the feedforward excitatory activity from bigrams to words. The fact that the Grainger and van Heuven (2004) model also implements inhibitory connectivity between bigrams and words led us to predict the pattern of results seen with nonword stimuli in the present study. Insertion nonwords contain more open-bigrams that are incompatible with the baseword than do repetition nonwords. Incompatible bigrams inhibit whole-word representations, such that the more incompatible bigrams there are in a target nonword the more the corresponding baseword will be inhibited, and the less likely this word will be perceived instead of the nonword. This therefore accounts for the observed greater ease in classifying insertion nonwords as such compared with the repetition nonwords.

As can be seen in Appendix A, the overlap model of Gomez et al. (2008) accurately accounts for the present findings. The parameters implemented in the simulations described by Pablo Gomez (Gomez, 2020) enabled repeated letters to overlap in the repetition nonwords, hence increasing the evidence that only one of these letters is present, and therefore increasing similarity with the baseword. Furthermore, the same mechanism can also account for the inhibitory effects of letter repetition found with word stimuli (Schoonbaert & Grainger, 2004; Trifonova & Adelman, 2019). Crucially, the overlap model also captures the monotonic decrease in repetition effects with distance seen in inverse efficiency scores in Experiment 2. On the other hand, the pattern of effects found in Experiment 2 is incompatible with Grainger and van Heuven’s version of open-bigram coding (see Appendix A). However, Grainger and van Heuven (2004) did suggest that imposing a strict limit on the maximal distance for open-bigram representations was clearly an over-simplification, and a scheme where bigrams are weighted by distance would be a more viable solution. The results of Experiment 2 are in line with this conjecture. Such a version of open-bigram coding had already been proposed by Whitney (2001), and further support for this approach was provided by Hannagan and Grainger (2012).

Finally, we acknowledge that it is possible that the letter-repetition effects found with words (Schoonbaert & Grainger, 2004; Trifonova & Adelman, 2019) and with nonwords (the present study) might not reflect mechanisms involved in orthographic processing, but might be driven by some form of spatial repetition blindness (RB: e.g., Kanwisher, 1991; Luo & Caramazza, 1996). If the second occurrence of a repeated letter was suppressed in some way, then this would make it harder to identify words with letter repetitions, and it would make the repeated letter nonwords look like their baseword. Here, it is important to note that Kanwisher (1991) investigated spatial RB with different kinds of stimuli – letters, symbols, and color patches – and found the effects to be quite similar for the different types of stimuli, hence pointing to a relatively low-level perceptual locus of the phenomenon. Furthermore, RB effects (both sequential and spatial) require quite short stimulus exposures (e.g., Kanwisher, 1991; Mozer, 1989), and spatial (simultaneous) RB is less pronounced than sequential RB (Kanwisher, 1991; Luo & Caramazza, 1996). This evidence points to a limitation in the ability to sequentially allocate attention to the different items as the main source of RB. This is therefore a very different mechanism compared with the kind of parallel orthographic processing typically assumed to operate during visual word recognition (e.g., Adelman, Marquis, & Sabatos-DeVito, 2010; Grainger, 2018).

In order to confirm the orthographic locus of letter-repetition effects, future research could compare repetition effects with different kinds of stimuli. This is possible using the same-different matching task, or the match-to-sample task used by (Gomez et al., 2008). In their Experiment 4, Gomez et al. (2008) found that accuracy dropped significantly when the target contained a letter repetition. It will be important to know whether such repetition effects in a relatively low-level task are of comparable magnitude for stimuli such as digits and symbols. The open-bigram account of letter-repetition effects predicts that the effects should be greater for letter stimuli, in the same manner as transposition effects in same-different matching (Duñabetia, Dimitropoulou, Grainger, Hernández, & Carreiras, 2012; Massol, Duñabetia, Carreiras, & Grainger, 2013; see Grainger & Hannagan, 2014, for a review). On the other hand, the overlap model, which implements a generic order-encoding mechanism, predicts that similar repetition effects should be observed for different types of stimuli. This offers an interesting avenue for future research aiming at testing these different accounts of non-adjacent letter-repetition effects.

## Appendix A

Predictions of three letter-position coding schemes tested with the stimuli of Experiments 1 and 2 using Colin Davis’ Match Calculator http://www.pc.rhul.ac.uk/staff/c.davis/utilities/matchcalc/index.htm for the SOLAR (spatial coding) model (Davis, 2010) and the binary Open Bigram model (Grainger & van Heuven, 2004), and the values provided by Pablo Gomez for the Overlap model (Gomez, 2020). Significant t-values are in bold, and negative values indicate differences in the incorrect direction

**Table:**

**Experiment 1**					
Model		Mean (SD)		t-test	
		Repetition	Insertion	Difference	*t*
SOLAR		0.914 (0.03)	0.922 (0.03)	−0.008	−**4.23**
Open Bigram		0.908 (0.05)	0.879 (0.05)	0.029	**9.45**
Overlap		3.052 (0.27)	3.000 (0.27)	0.052	**10.75**
**Experiment 2**					
Model	Distance	Mean (SD)			t-test
		Repetition	Insertion	Difference	*t*
SOLAR	1	0.900 (0.02)	0.900 (0.02)	0	0
	2	0.902 (0.01)	0.907 (0.02)	−0.005	−**2.25**
	3	0.935 (0.02)	0.935 (0.01)	0	0
	4	0.956 (0.02)	0.956 (0.01)	0	0
Opea Bigram	1	0.905 (0.03)	0.848 (0.04)	0.057	**15.12**
	2	0.902 (0.03)	0.849 (0.04)	0.053	**18.96**
	3	0.865 (0.03)	0.865 (0.03)	0	0
	4	0.917 (0.03)	0.917 (0.03)	0	0
Overlap	1	3.092 (0.08)	2.939 (0.08)	0.153	**141.2**
	2	2.990 (0.07)	2.928 (0.07)	0.062	**21.20**
	3	2.912 (0.07)	2.898 (0.07)	0.014	**13.39**
	4	2.892 (0.12)	2.890 (0.12)	0.002	**8.498**

## Appendix B

Nonword stimuli tested in Experiment 1, and the basewords from which they were generated.

**Table:**

**Baseword**	**Repetition**	**Insertion**
adjudant	adjudtant	adjudlant
adolescence	adsolescence	admolescence
adversaire	adversairse	adversairle
ambition	ambintion	ambiction
amertume	amertumte	amertumse
baignoire	baignoirne	baignoirge
banlieue	banlienue	banlietue
blancheur	blanchreur	blanchteur
boulevard	bouledvard	bouletvard
boutique	boubtique	boultique
camarade	camadrade	camatrade
campagne	campcagne	campragne
caoutchouc	catoutchouc	camoutchouc
capitaine	capitnaine	capitgaine
catastrophe	catrastrophe	catlastrophe
cauchemar	cauchermar	cauchetmar
cercueil	cerclueil	cercnueil
certitude	certitrude	certitsude
chevalier	chevaliver	chevaliger
chevelure	cheverlure	chevetlure
circonstance	circornstance	circognstance
compagnie	compnagnie	compragnie
compagnon	comnpagnon	comspagnon
comptoir	comptroir	comptloir
concierge	concinerge	conciberge
conclusion	consclusion	contclusion
condition	contdition	consdition
confiance	conficance	confirance
confusion	confunsion	confugsion
conscience	conscinence	conscidence
conviction	contviction	condviction
couvercle	couvlercle	couvdercle
couverture	coutverture	coulverture
discipline	discilpline	discimpline
domicile	domilcile	domircile
empereur	ermpereur	elmpereur
escalier	escarlier	escaplier
exercice	exercirce	exercince
existence	existnence	existpence
fauteuil	fautfeuil	fautreuil
fermeture	fertmeture	ferbmeture
fonction	foncticon	fonctibon
fontaine	fontaitne	fontaigne
gendarme	endmarme	gendsarme
grandeur	grandreur	grandleur
habitude	habitbude	habitrude
harmonie	harmonrie	harmondie
histoire	histroire	histloire
ignorance	ignorgance	ignormance
individu	indinvidu	indirvidu
indulgence	indgulgence	indmulgence
instinct	instninct	instrinct
instrument	instrumtent	instrumpent
jalousie	jalsousie	jalrousie
journaliste	journasliste	journacliste
lendemain	lendemdain	lendempain
lieutenant	lienutenant	lierutenant
longueur	longuenur	longuepur
mensonge	mensgonge	menslonge
ministre	mitnistre	midnistre
monsieur	monsrieur	monstieur
montagne	mogntagne	morntagne
mouchoir	mohuchoir	moluchoir
moustache	moucstache	mounstache
nostalgie	nostnalgie	nostralgie
ouverture	outverture	oumverture
patience	pantience	paftience
peinture	petinture	peminture
perfection	pertfection	permfection
poitrine	proitrine	ploitrine
prestige	prestrige	prestnige
principe	prinpcipe	prindcipe
profondeur	pronfondeur	protfondeur
promenade	promednade	promelnade
province	pronvince	prodvince
prudence	prundence	prusdence
quartier	quartrier	quartlier
question	questison	questihon
religion	regligion	remligion
retraite	retratite	retralite
revolver	relvolver	remvolver
sacrifice	scacrifice	slacrifice
scandale	scandcale	scandrale
sensation	sentsation	senbsation
signature	snignature	spignature
solution	solustion	soluction
spectacle	spectlacle	spectracle
sympathie	spympathie	slympathie
tabouret	tarbouret	tambouret
tendance	tendtance	tendjance
tentative	tentantive	tentartive
trahison	trahrison	trahmison
tribunal	triburnal	tribudnal
troupeau	trotupeau	trofupeau
vendredi	verndredi	vemndredi
vengeance	vengveance	vengdeance
victoire	victroire	victmoire
vingtaine	vingtnaine	vingtraine
violence	violvence	violdence
vocation	vocantion	vocastion

## Appendix C

Nonword stimuli tested in Experiment 2 and the basewords from which they were generated.

**Table:**

**Baseword**	**Repetition**	**Insertion**	**Distance**
abstenir	abstsenir	abstmenir	1
aiguiser	aigusiser	aiguliser	1
ambition	ambibtion	ambirtion	1
artifice	artrifice	artnifice	1
aviateur	avivateur	avicateur	1
banquier	banqnuier	banqluier	1
blanchir	blanhchir	blanpchir	1
calvaire	calvlaire	calvpaire	1
carabine	cararbine	caratbine	1
cercueil	cercrueil	cercnueil	1
chanteur	chahnteur	chamnteur	1
cheminer	chehminer	chetminer	1
conjugal	conjnugal	conjrugal	1
descente	descnente	descrente	1
diminuer	dimimnuer	dimisnuer	1
division	divivsion	divirsion	1
douzaine	douzanine	douzabine	1
escadron	escardron	escapdron	1
estomper	estotmper	estohmper	1
exclusif	exclsusif	excltusif	1
expulser	expuplser	exputlser	1
farouche	faroruche	faroduche	1
faubourg	fauborurg	faubonurg	1
flambeau	flalmbeau	flarmbeau	1
fragment	frargment	fralgment	1
gracieux	grarcieux	gratcieux	1
hargneux	hargrneux	harglneux	1
imaginer	imamginer	imacginer	1
infliger	inflgiger	inflpiger	1
invoquer	invovquer	invotquer	1
lamenter	lamemnter	lamesnter	1
lumineux	lumimneux	lumitneux	1
migraine	migranine	migradine	1
ministre	mininstre	minilstre	1
novembre	novevmbre	novetmbre	1
paisible	paisilble	paisirble	1
patauger	patatuger	patanuger	1
plaindre	plalindre	plabindre	1
poitrine	poitrnine	poitrzine	1
prestige	prerstige	prebstige	1
purement	purerment	puresment	1
relation	relaltion	relaption	1
rudement	rudedment	redelment	1
scandale	scandlale	scandfale	1
signaler	signgaler	signcaler	1
sourdine	sourdnine	sourdcine	1
suicider	suicdider	suiclider	1
trahison	trahsison	trahmison	1
utiliser	utitliser	utinliser	1
visiteur	visisteur	visinteur	1
absenter	absenster	absencter	2
alentour	alelntour	alepntour	2
analyser	anaslyser	anamlyser	2
astiquer	astisquer	astinquer	2
aviation	aviavtion	aviartion	2
baptiser	bapstiser	bapltiser	2
camarade	camadrade	camanrade	2
cantique	cantinque	cantirque	2
cartable	carbtable	carstable	2
champion	chamhpion	chamspion	2
chantier	chanhtier	chanptier	2
composer	compomser	compotser	2
consoler	consonler	consogler	2
cuisiner	cuisinser	cuisinder	2
destiner	destisner	destibner	2
disciple	discisple	discirple	2
doctrine	doctrcine	doctrline	2
durement	durenment	duresment	2
escalier	escalcier	escalpier	2
euphorie	euphoprie	euphodrie	2
exigence	exingence	exitgence	2
extasier	extastier	extaslier	2
fasciner	fascisner	fascitner	2
flanquer	flanlquer	flantquer	2
gaiement	gaienment	gaierment	2
habitude	habitbude	habitrude	2
harmonie	harmornie	harmotnie	2
indiquer	indinquer	indilquer	2
insolite	insotlite	insorlite	2
ironique	ironrique	ironsique	2
magazine	maganzine	magarzine	2
modestie	modtestie	modrestie	2
monsieur	monsineur	monsileur	2
munition	munitnion	munitrion	2
obstacle	obstlacle	obstracle	2
pancarte	panctarte	pancharte	2
patience	paticence	patirence	2
planquer	planlquer	plansquer	2
poliment	polinment	polidment	2
province	pronvince	prolvince	2
pyramide	pyradmide	pyralmide	2
religion	religlion	religsion	2
sagement	sagenment	sagerment	2
scrupule	scrulpule	scrumpule	2
solitude	soliltude	solirtude	2
soulager	souglager	soutlager	2
syndicat	syndincat	syndircat	2
triangle	trigangle	trisangle	2
victoire	victroire	victboire	2
vocation	vocatcion	vocatrion	2
adorable	adolrable	adocrable	3
anecdote	anecdnote	anecdrote	3
atomique	atomitque	atomilque	3
auditeur	auditedur	auditelur	3
basculer	basculser	basculner	3
boutique	boqutique	bolutique	3
bricoler	bricorler	bricotler	3
campagne	camnpagne	camrpagne	3
cendrier	cendriner	cendriver	3
chandail	chandhail	chandmail	3
chapelet	chapehlet	chaperlet	3
combiner	combinmer	combinder	3
comptoir	comptomir	comptolir	3
consumer	consumner	consumper	3
devancer	decvancer	delvancer	3
discuter	discutser	discutler	3
document	docnument	docrument	3
employer	employper	employter	3
encolure	encolnure	encoldure	3
escargot	escarsgot	escarmgot	3
examiner	exnaminer	expaminer	3
fabuleux	fabulebux	fabulemux	3
fatigant	fatnigant	fatrigant	3
fixement	fixnement	fixlement	3
gendarme	gendarnme	gendartme	3
habituel	habitubel	habiturel	3
histoire	histoisre	histoinre	3
insulter	insulnter	insulpter	3
jalousie	jalouslie	jaloustie	3
limonade	limdonade	limronade	3
logement	lognement	logrement	3
maternel	manternel	masternel	3
modifier	modifider	modifiler	3
naviguer	naviguver	naviguler	3
obstiner	obstibner	obstigner	3
paniquer	paniquner	paniquher	3
paquebot	pabquebot	parquebot	3
plaintif	plainltif	plainstif	3
pleuvoir	pleuvloir	pleuvboir	3
ponctuer	ponctuner	ponctuder	3
prudence	prucdence	prusdence	3
quatorze	quaztorze	quaptorze	3
revanche	recvanche	remvanche	3
sculpter	sculpcter	sculphter	3
solution	solutilon	solutiron	3
soulever	sovulever	sobulever	3
toujours	tourjours	tounjours	3
tribunal	triburnal	tribusnal	3
violence	vioclence	viorlence	3
vraiment	vraniment	vradiment	3
agricole	agricogle	agricotle	4
ambiance	amcbiance	amtbiance	4
apitoyer	apitoyper	apitoyder	4
argument	arngument	arpgument	4
aveugler	alveugler	adveugler	4
balancer	bcalancer	bhalancer	4
bestiole	bestiolse	bestiolde	4
bracelet	bracelret	bracelnet	4
caniveau	caniveanu	canivealu	4
caravane	canravane	catravane	4
chantage	chantahge	chantarge	4
charogne	chnarogne	chbarogne	4
conduire	cornduire	cojnduire	4
craintif	craintrif	craintlif	4
croisade	croisarde	croisande	4
dimanche	dihmanche	dirmanche	4
disputer	ditsputer	dinsputer	4
domicile	dolmicile	dotmicile	4
enfilade	enfilande	enfilarde	4
enjamber	enjambner	enjambler	4
esquiver	evsquiver	elsquiver	4
exclamer	exclamxer	exclamter	4
fatiguer	fatiguetr	fatiguenr	4
flagrant	flagralnt	flagrasnt	4
fraction	fractiron	fractibon	4
glorieux	glorielux	gloriedux	4
habituer	habituebr	habituenr	4
investir	investnir	investlir	4
jardinet	jardinert	jardinept	4
jugement	jungement	jucgement	4
longueur	longueunr	longueutr	4
machinal	mnachinal	msachinal	4
milicien	milicieln	miliciern	4
moquerie	mroquerie	mnoquerie	4
narquois	narquoirs	narquoips	4
objecter	objectber	objectler	4
originel	originrel	origindel	4
paradoxe	paxradoxe	pamradoxe	4
peinture	perinture	pedinture	4
poignard	porignard	pofignard	4
position	positiosn	positiorn	4
rigolade	ridgolade	ringolade	4
salement	sanlement	sadlement	4
sanglier	sanglienr	sanglietr	4
somnoler	slomnoler	stomnoler	4
soutenir	snoutenir	scoutenir	4
tourment	tonurment	tolurment	4
tropical	tropicral	tropicmal	4
troupeau	trouperau	troupebau	4
virginie	virginire	virginide	4

## Footnotes

1 We dedicate this work to the memory of Carol Whitney (1963–2019). We only got to know about Carol’s untimely passing-away when we tried to contact her while working on this article. Carol was a pioneer in the field of what we now call “orthographic processing,” and her early contribution with the SERIOL model (Whitney, 2001) was of utmost importance. Carol was a great mind and a beautiful person.

2 The term “principled” is used here to underline the fact that open-bigram coding was not invented to account for letter-repetition effects or for transposed-letter effects (Grainger & Whitney, 2004). These effects are true predictions derived from the core mechanism of this coding scheme. Core mechanisms are distinguished from parameters such as the maximum distance between two non-contiguous letters, and whether or not bigrams are weighted by distance or position (see Hannagan & Grainger, 2012, for an analysis of such parameters within the more general framework of String kernels).

3 Open-bigram coding is only one means to encode letter order information within a more general framework for visual word recognition that also allows for more precise order encoding (Grainger, 2018; Grainger & Ziegler, 2011). The level of precision is determined by the nature of the task (silent reading vs. reading aloud) and the discriminability of the word with respect to all other words in the lexicon (more precision with low discriminability: Meade, Mahnich, Holcomb, & Grainger, 2020).

4 In Appendix A we provide predictions from different letter-position coding schemes. Note, nevertheless, that the experiments were entirely motivated by an open-bigram coding account of prior observations of letter-repetition effects in words.

5 OLD20 measures the orthographic Levenshtein distance between a given letter sequence and all existing words. It takes the 20 closest (smallest) measures and outputs the average of those values.
